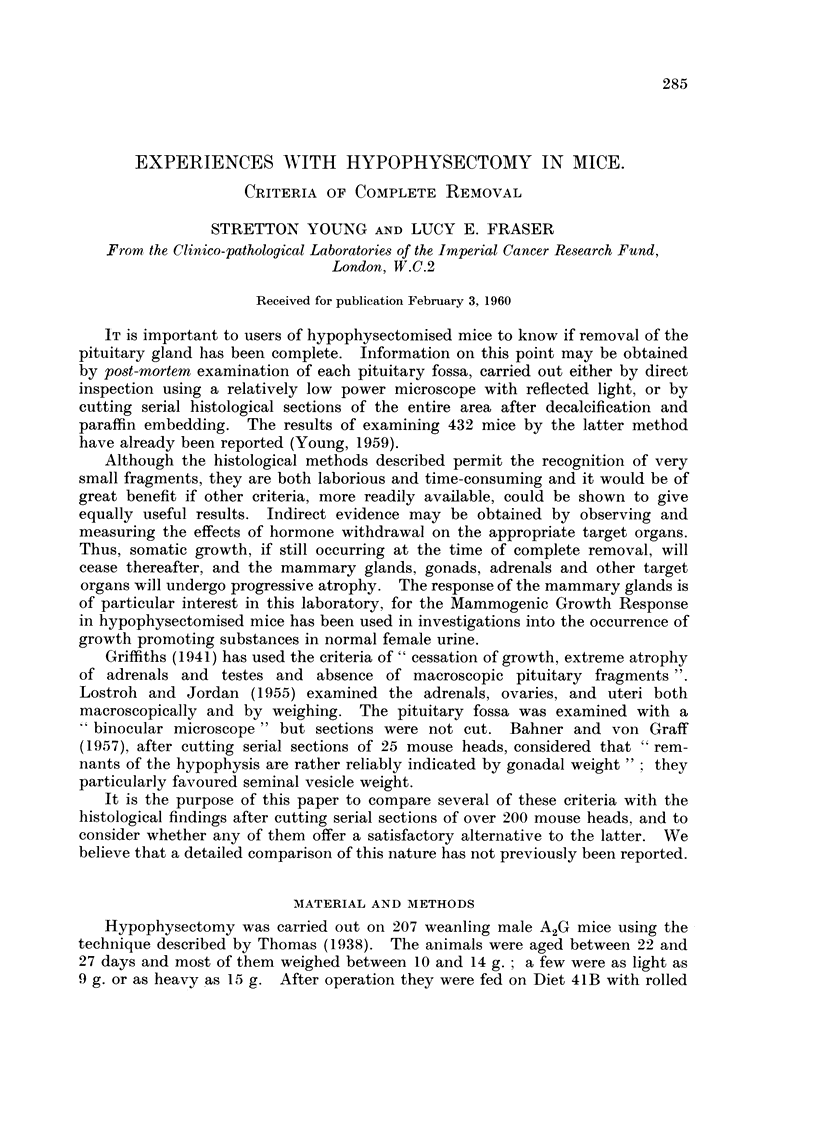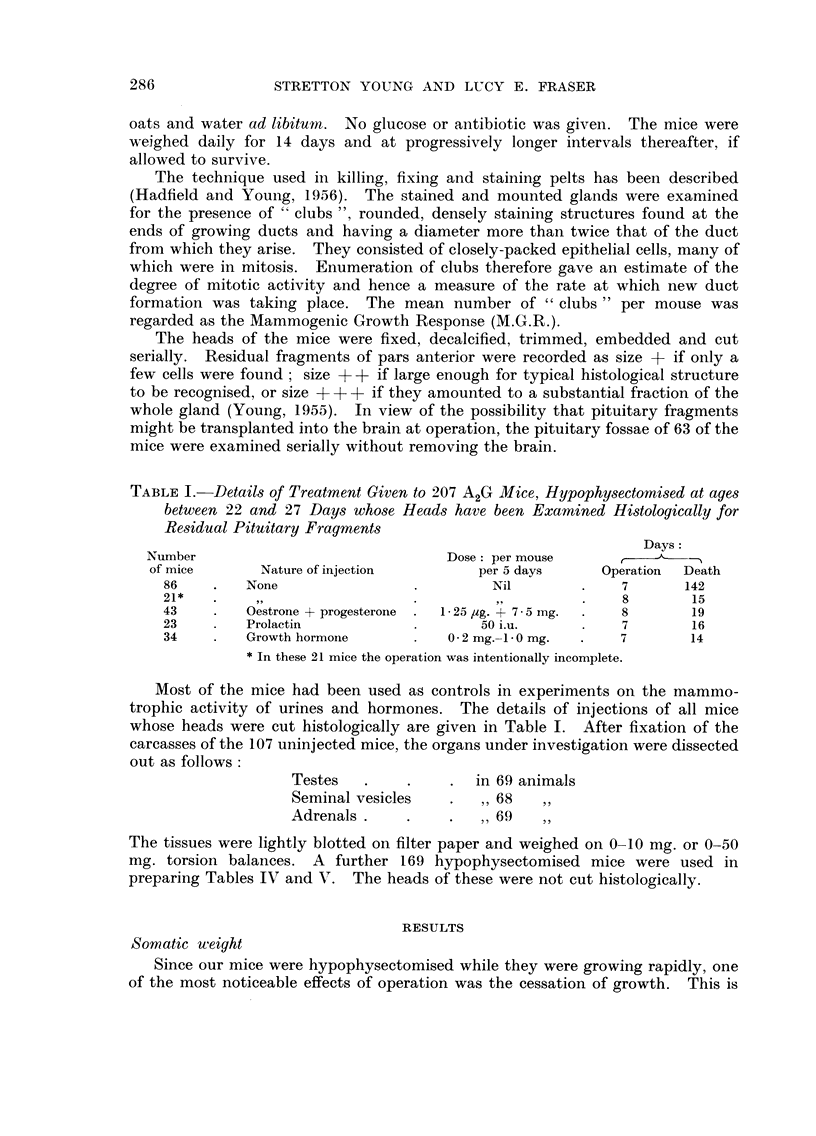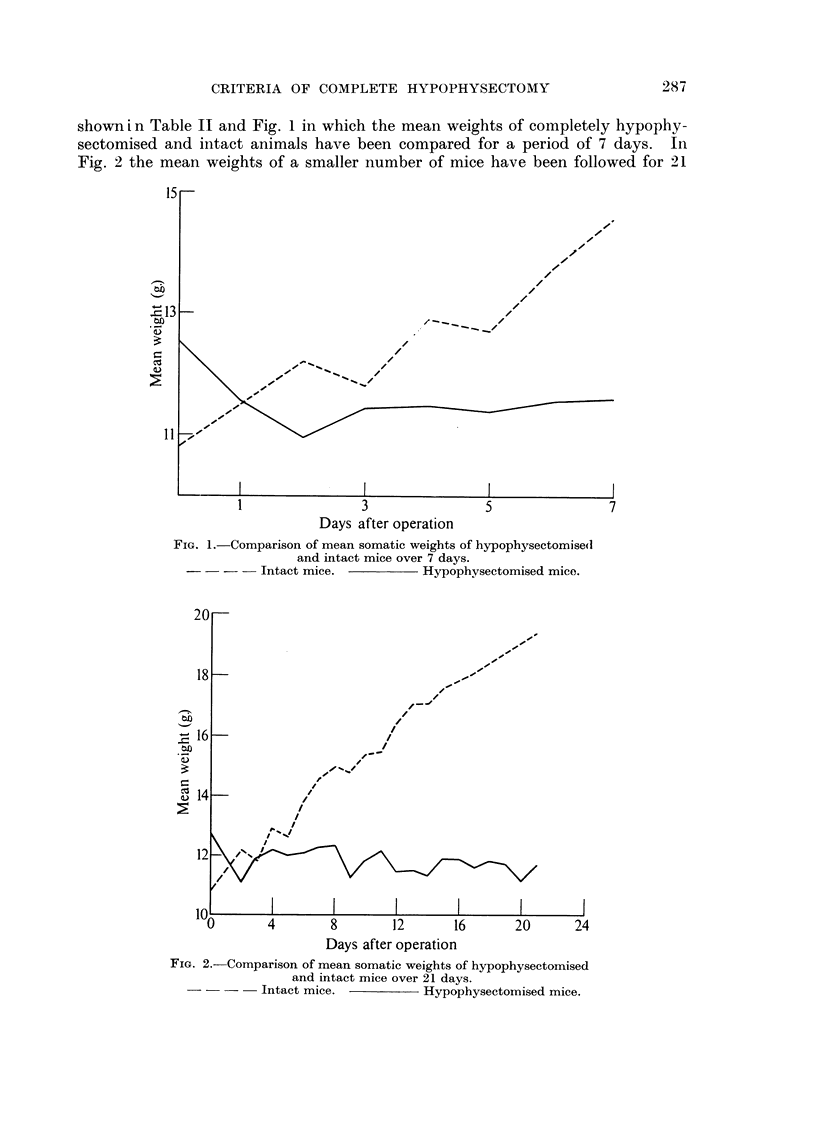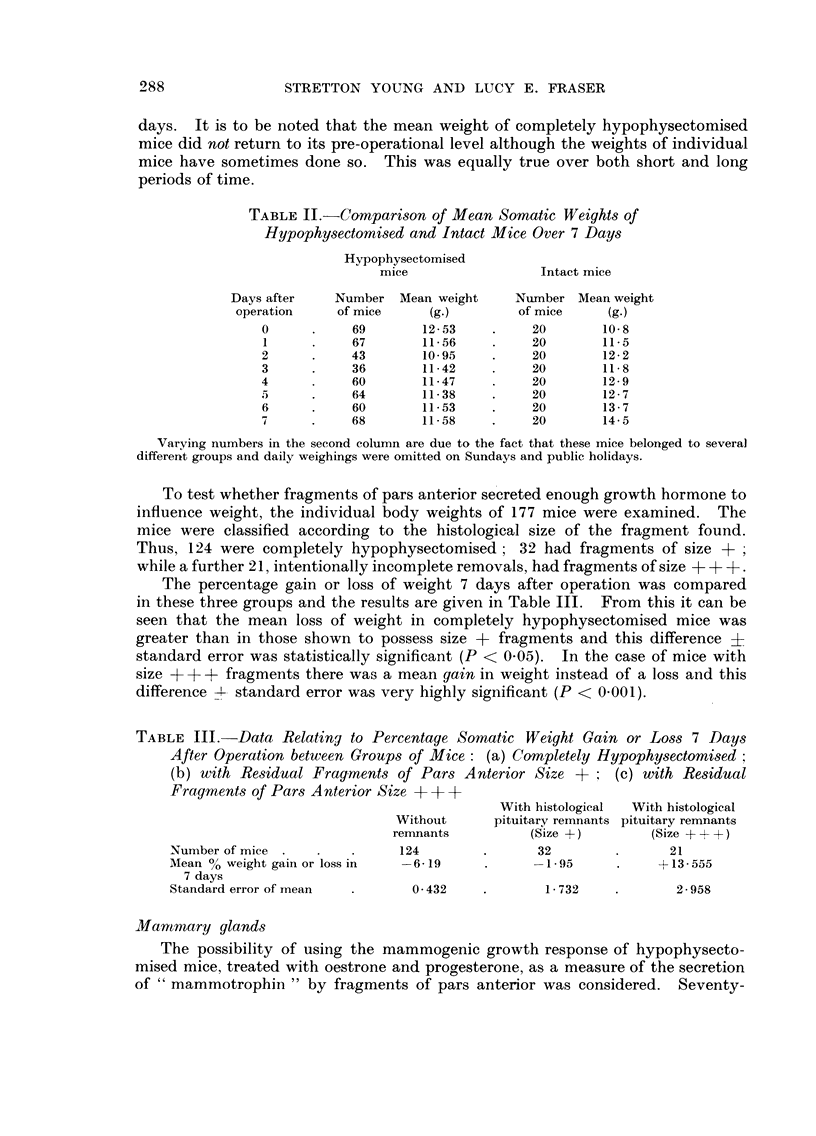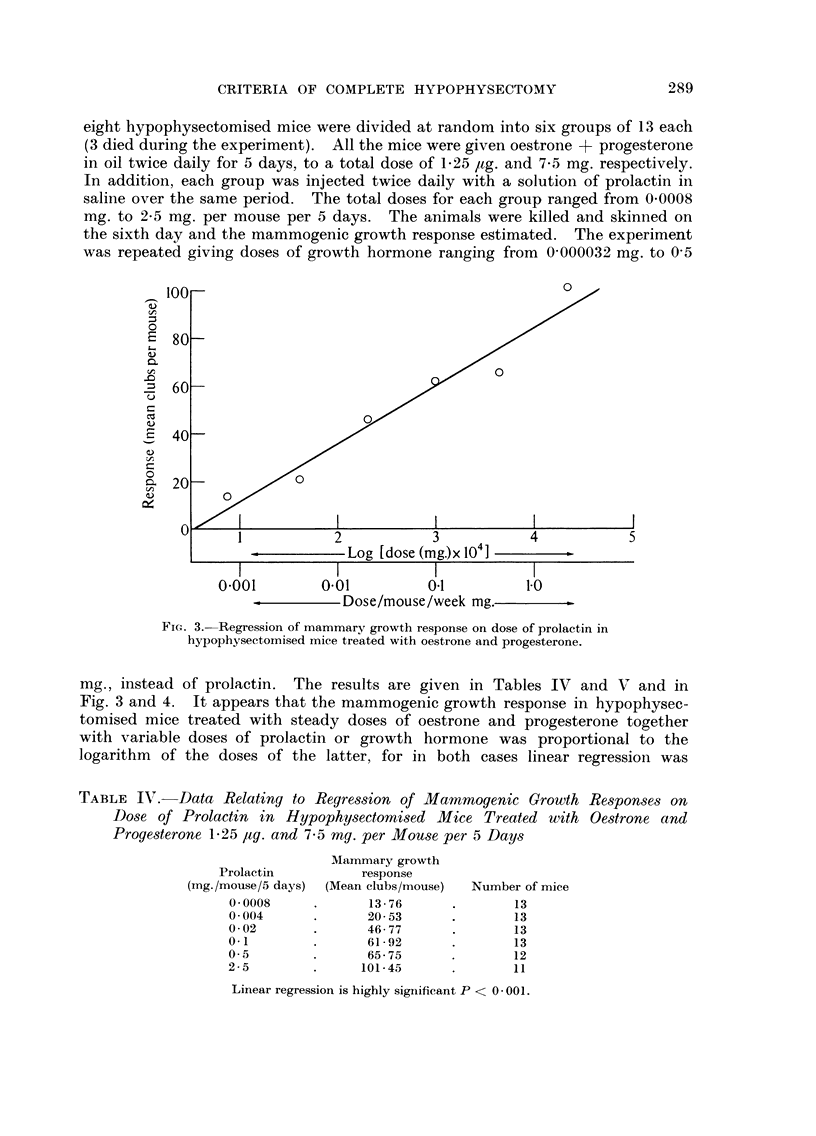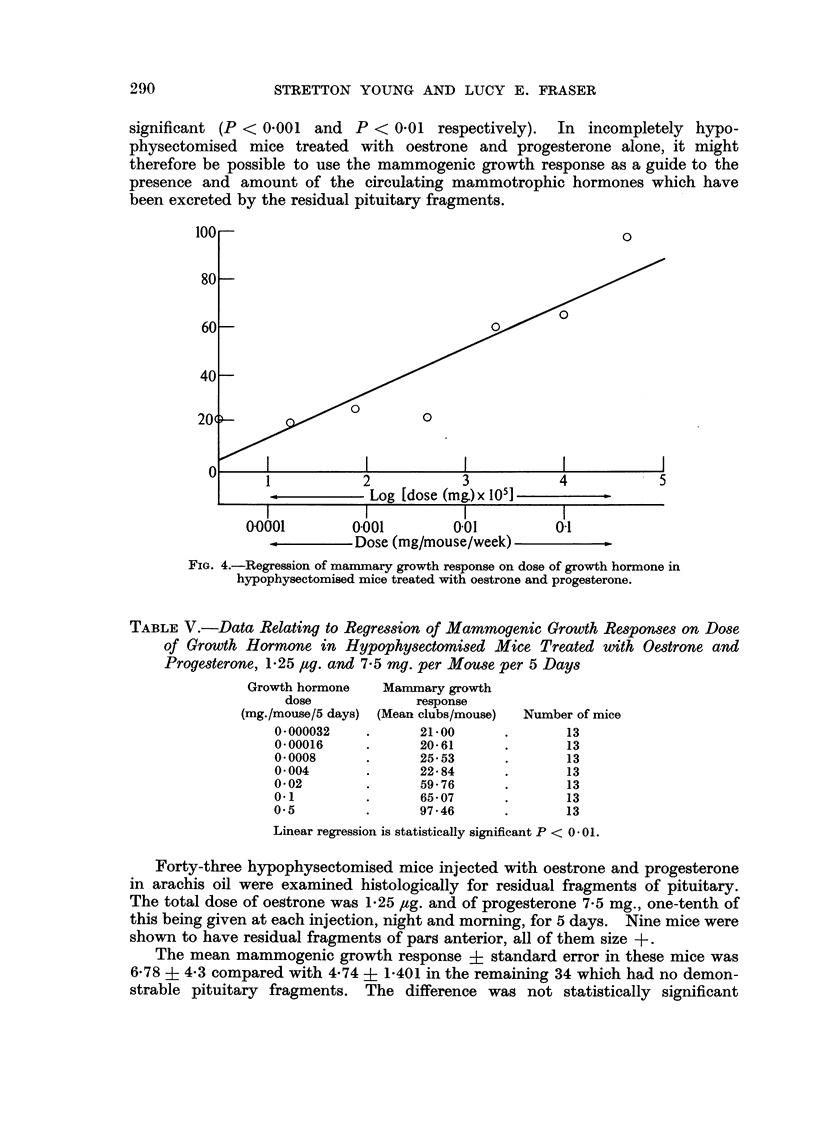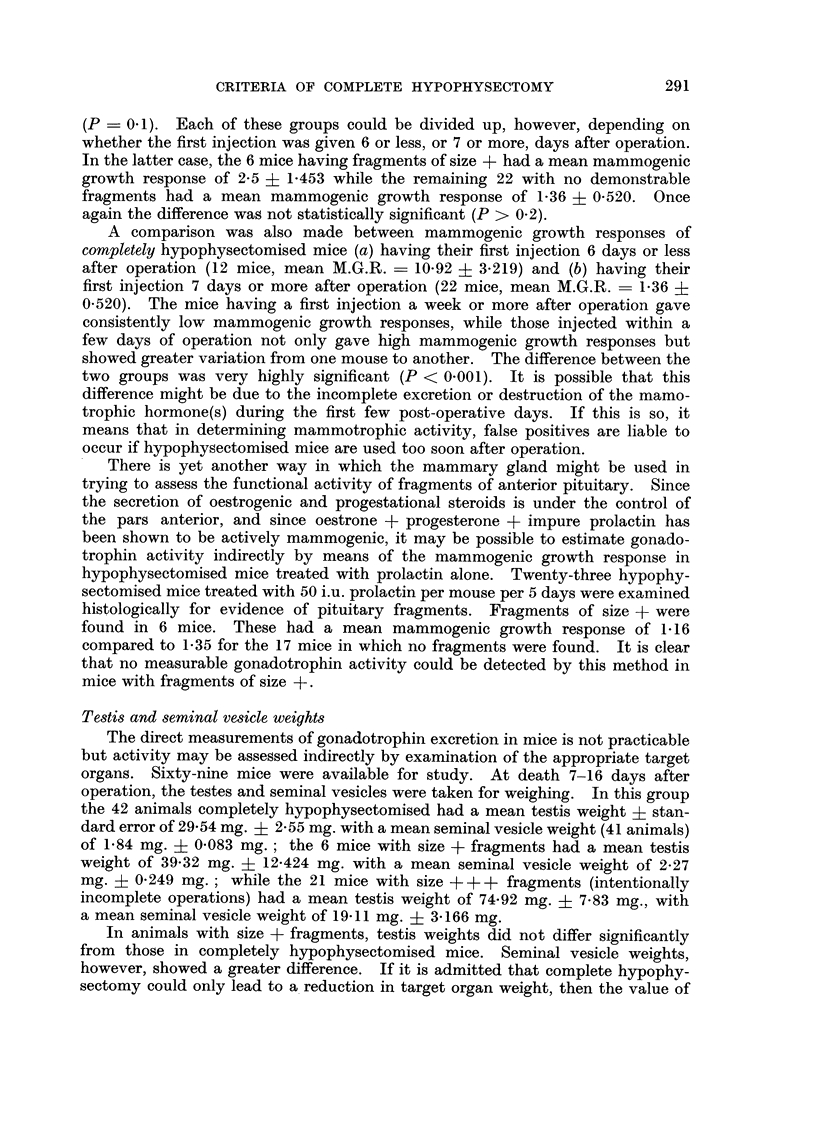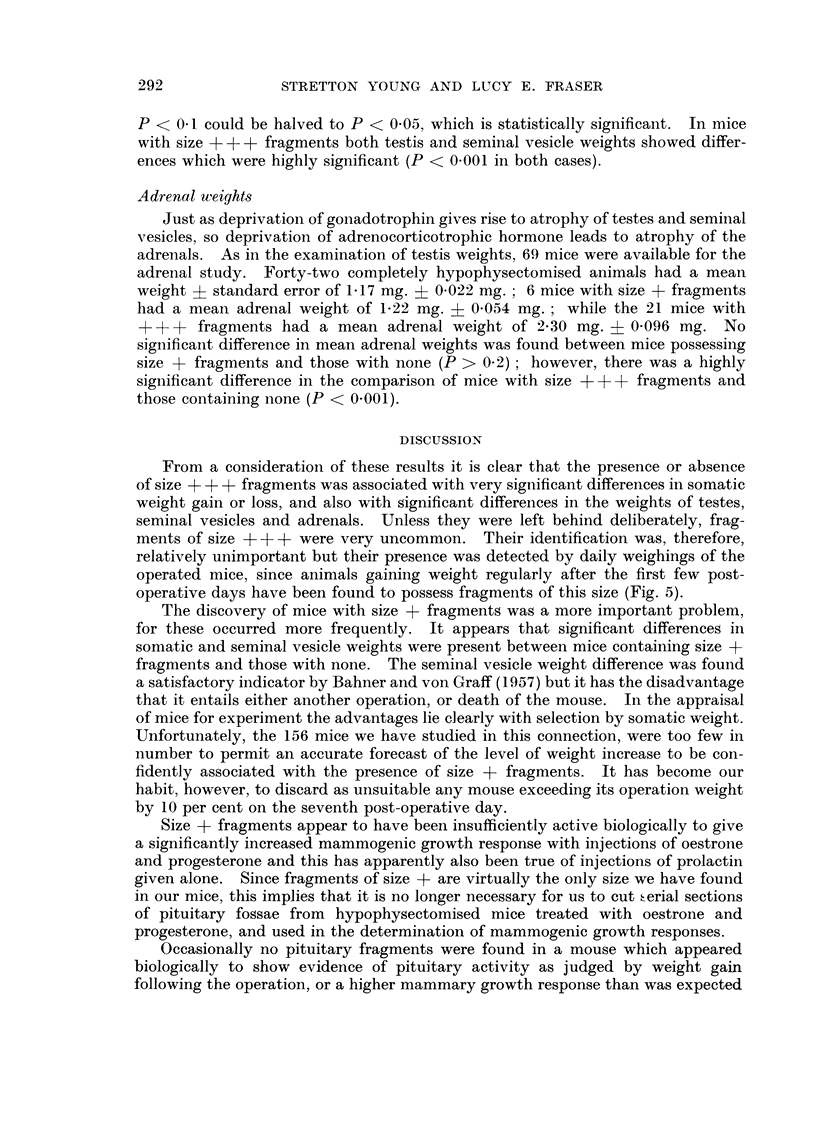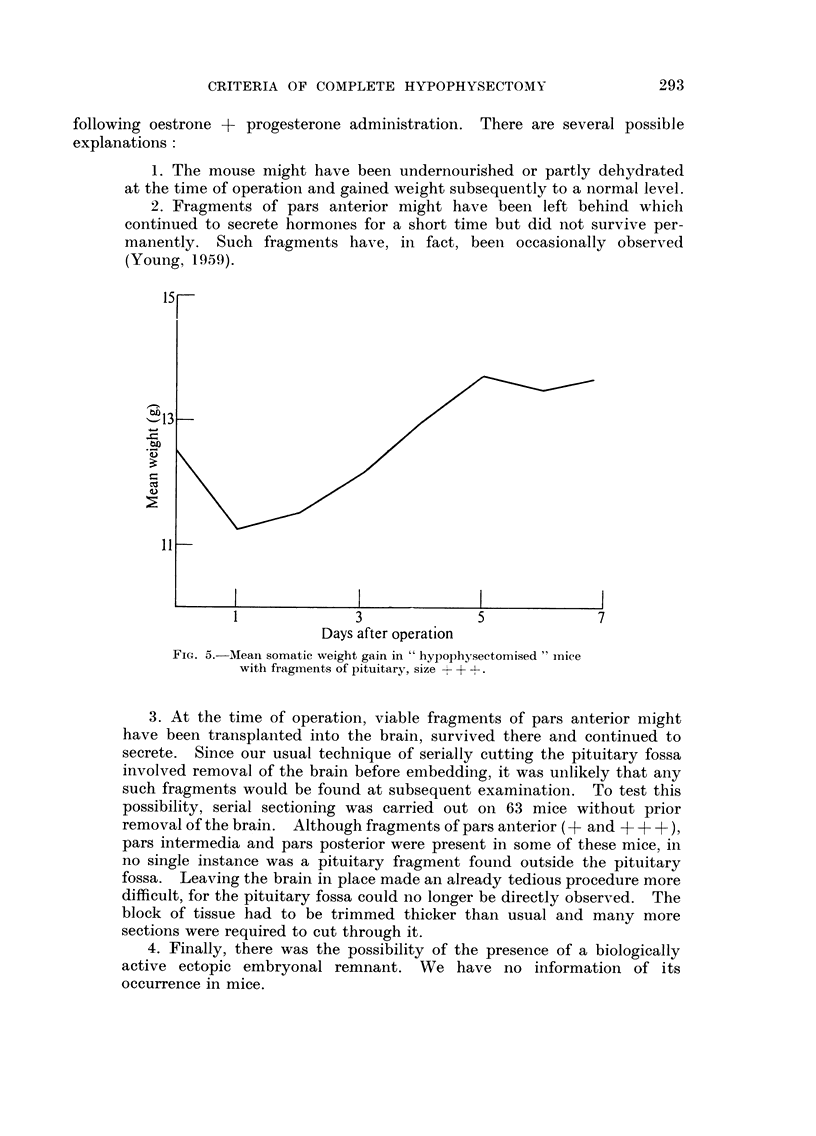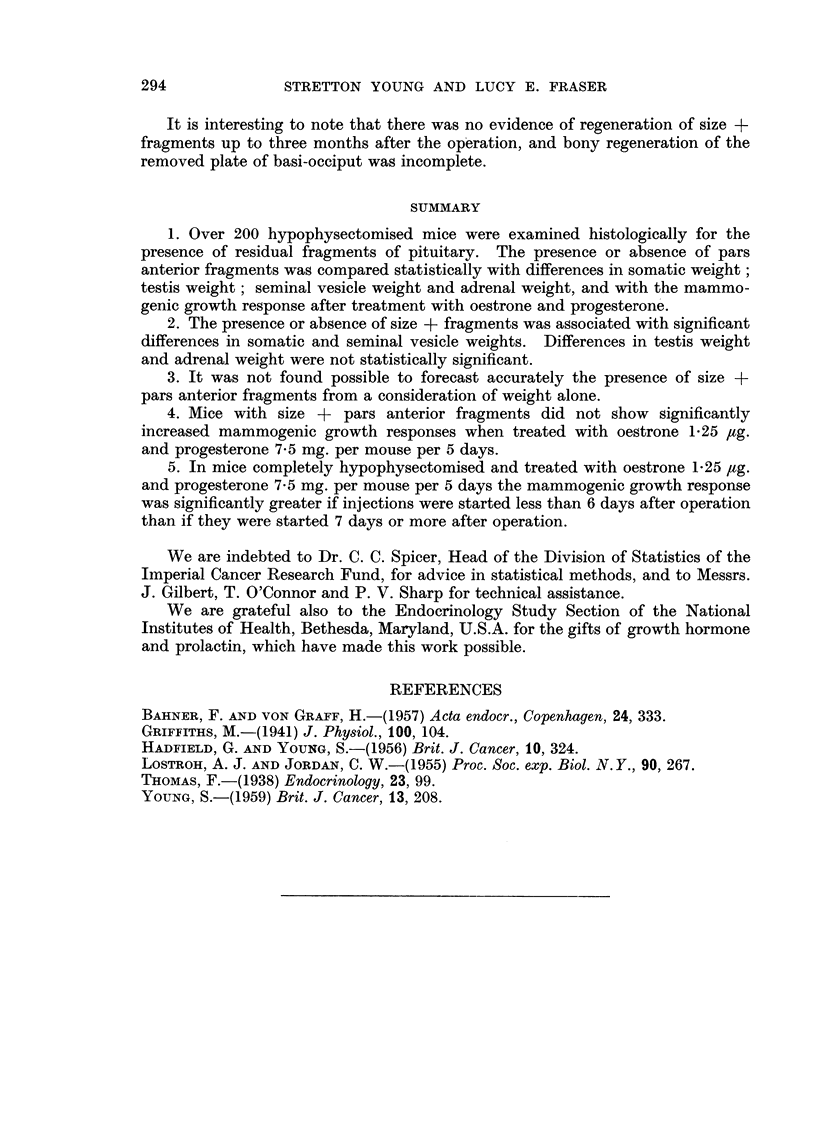# Experiences with Hypophysectomy in Mice

**DOI:** 10.1038/bjc.1960.33

**Published:** 1960-06

**Authors:** Stretton Young, Lucy E. Fraser


### EXPERIENCES WITH HYPOPHYSECTOMY IN MICE.

### Criteria of Complete Removal

STRETTON YOUNG and LUCY E. FRASER

*From the Clinico-pathological Laboratories of the Imperial Cancer Research Fund, London, W.C.2*

Received for publication February 3, 1960

It is important to users of hypophysectomised mice to know if removal of the pituitary gland has been complete. Information on this point may be obtained by *post-mortem* examination of each pituitary fossa, carried out either by direct inspection using a relatively low power microscope with reflected light, or by cutting serial histological sections of the entire area after decalcification and paraffin embedding. The results of examining 432 mice by the latter method have already been reported (Young, 1959).

Although the histological methods described permit the recognition of very small fragments, they are both laborious and time-consuming and it would be of great benefit if other criteria, more readily available, could be shown to give equally useful results. Indirect evidence may be obtained by observing and measuring the effects of hormone withdrawal on the appropriate target organs. Thus, somatic growth, if still occurring at the time of complete removal, will cease thereafter, and the mammary glands, gonads, adrenals and other target organs will undergo progressive atrophy. The response of the mammary glands is of particular interest in this laboratory, for the Mammogenic Growth Response in hypophysectomised mice has been used in investigations into the occurrence of growth promoting substances in normal female urine.

Griffiths (1941) has used the criteria of " cessation of growth, extreme atrophy of adrenals and testes and absence of macroscopic pituitary fragments ". Lostroh and Jordan (1955) examined the adrenals, ovaries, and uteri both macroscopically and by weighing. The pituitary fossa was examined with a " binocular microscope " but sections were not cut. Bahner and von Graff (1957), after cutting serial sections of 25 mouse heads, considered that " remnants of the hypophysis are rather reliably indicated by gonadal weight " ; they particularly favoured seminal vesicle weight.

It is the purpose of this paper to compare several of these criteria with the histological findings after cutting serial sections of over 200 mouse heads, and to consider whether any of them offer a satisfactory alternative to the latter. We believe that a detailed comparison of this nature has not previously been reported.

### MATERIAL AND METHODS

Hypophysectomy was carried out on 207 weanling male $A_2G$ mice using the technique described by Thomas (1938). The animals were aged between 22 and 27 days and most of them weighed between 10 and 14 g. ; a few were as light as 9 g. or as heavy as 15 g. After operation they were fed on Diet 41B with rolled oats and water *ad libitum*. No glucose or antibiotic was given. The mice were weighed daily for 14 days and at progressively longer intervals thereafter, if allowed to survive.

The technique used in killing, fixing and staining pelts has been described (Hadfield and Young, 1956). The stained and mounted glands were examined for the presence of " clubs ", rounded, densely staining structures found at the ends of growing ducts and having a diameter more than twice that of the duct from which they arise. They consisted of closely-packed epithelial cells, many of which were in mitosis. Enumeration of clubs therefore gave an estimate of the degree of mitotic activity and hence a measure of the rate at which new duct formation was taking place. The mean number of " clubs " per mouse was regarded as the Mammogenic Growth Response (M.G.R.).

The heads of the mice were fixed, decalcified, trimmed, embedded and cut serially. Residual fragments of pars anterior were recorded as size + if only a few cells were found ; size ++ if large enough for typical histological structure to be recognised, or size +++ if they amounted to a substantial fraction of the whole gland (Young, 1955). In view of the possibility that pituitary fragments might be transplanted into the brain at operation, the pituitary fossae of 63 of the mice were examined serially without removing the brain.

TABLE I.—*Details of Treatment Given to 207 $A_2G$ Mice, Hypophysectomised at ages between 22 and 27 Days whose Heads have been Examined Histologically for Residual Pituitary Fragments*

| Number of mice | Nature of injection | Dose : per mouse per 5 days | Days : Operation | Days : Death |
|---|---|---|---|---|
| 86 | None | Nil | 7 | 142 |
| 21* | ,, | ,, | 8 | 15 |
| 43 | Oestrone + progesterone | 1·25 μg. + 7·5 mg. | 8 | 19 |
| 23 | Prolactin | 50 i.u. | 7 | 16 |
| 34 | Growth hormone | 0·2 mg.–1·0 mg. | 7 | 14 |

* In these 21 mice the operation was intentionally incomplete.

Most of the mice had been used as controls in experiments on the mammotrophic activity of urines and hormones. The details of injections of all mice whose heads were cut histologically are given in Table I. After fixation of the carcasses of the 107 uninjected mice, the organs under investigation were dissected out as follows :

| | |
|---|---|
| Testes . . . | in 69 animals |
| Seminal vesicles . | ,, 68 ,, |
| Adrenals . . . | ,, 69 ,, |

The tissues were lightly blotted on filter paper and weighed on 0–10 mg. or 0–50 mg. torsion balances. A further 169 hypophysectomised mice were used in preparing Tables IV and V. The heads of these were not cut histologically.

### RESULTS

### *Somatic weight*

Since our mice were hypophysectomised while they were growing rapidly, one of the most noticeable effects of operation was the cessation of growth. This is shown in Table II and Fig. 1 in which the mean weights of completely hypophysectomised and intact animals have been compared for a period of 7 days. In Fig. 2 the mean weights of a smaller number of mice have been followed for 21

FIG. 1.—Comparison of mean somatic weights of hypophysectomised and intact mice over 7 days.
— — — — Intact mice. ———— Hypophysectomised mice.

FIG. 2.—Comparison of mean somatic weights of hypophysectomised and intact mice over 21 days.
— — — — Intact mice. ———— Hypophysectomised mice.

days. It is to be noted that the mean weight of completely hypophysectomised mice did *not* return to its pre-operational level although the weights of individual mice have sometimes done so. This was equally true over both short and long periods of time.

TABLE II.—*Comparison of Mean Somatic Weights of Hypophysectomised and Intact Mice Over* 7 *Days*

| | Hypophysectomised mice | | Intact mice | |
|---|---|---|---|---|
| Days after operation | Number of mice | Mean weight (g.) | Number of mice | Mean weight (g.) |
| 0 | 69 | 12·53 | 20 | 10·8 |
| 1 | 67 | 11·56 | 20 | 11·5 |
| 2 | 43 | 10·95 | 20 | 12·2 |
| 3 | 36 | 11·42 | 20 | 11·8 |
| 4 | 60 | 11·47 | 20 | 12·9 |
| 5 | 64 | 11·38 | 20 | 12·7 |
| 6 | 60 | 11·53 | 20 | 13·7 |
| 7 | 68 | 11·58 | 20 | 14·5 |

Varying numbers in the second column are due to the fact that these mice belonged to several different groups and daily weighings were omitted on Sundays and public holidays.

To test whether fragments of pars anterior secreted enough growth hormone to influence weight, the individual body weights of 177 mice were examined. The mice were classified according to the histological size of the fragment found. Thus, 124 were completely hypophysectomised ; 32 had fragments of size + ; while a further 21, intentionally incomplete removals, had fragments of size + + +.

The percentage gain or loss of weight 7 days after operation was compared in these three groups and the results are given in Table III. From this it can be seen that the mean loss of weight in completely hypophysectomised mice was greater than in those shown to possess size + fragments and this difference ± standard error was statistically significant ($P < 0{\cdot}05$). In the case of mice with size + + + fragments there was a mean *gain* in weight instead of a loss and this difference ± standard error was very highly significant ($P < 0{\cdot}001$).

TABLE III.—*Data Relating to Percentage Somatic Weight Gain or Loss* 7 *Days After Operation between Groups of Mice* : (a) *Completely Hypophysectomised* ; (b) *with Residual Fragments of Pars Anterior Size* + ; (c) *with Residual Fragments of Pars Anterior Size* + + +

| | Without remnants | With histological pituitary remnants (Size +) | With histological pituitary remnants (Size + + +) |
|---|---|---|---|
| Number of mice | 124 | 32 | 21 |
| Mean % weight gain or loss in 7 days | −6·19 | −1·95 | +13·555 |
| Standard error of mean | 0·432 | 1·732 | 2·958 |

*Mammary glands*

The possibility of using the mammogenic growth response of hypophysectomised mice, treated with oestrone and progesterone, as a measure of the secretion of " mammotrophin " by fragments of pars anterior was considered. Seventyeight hypophysectomised mice were divided at random into six groups of 13 each (3 died during the experiment). All the mice were given oestrone + progesterone in oil twice daily for 5 days, to a total dose of 1·25 μg. and 7·5 mg. respectively. In addition, each group was injected twice daily with a solution of prolactin in saline over the same period. The total doses for each group ranged from 0·0008 mg. to 2·5 mg. per mouse per 5 days. The animals were killed and skinned on the sixth day and the mammogenic growth response estimated. The experiment was repeated giving doses of growth hormone ranging from 0·000032 mg. to 0·5

FIG. 3.—Regression of mammary growth response on dose of prolactin in hypophysectomised mice treated with oestrone and progesterone.

mg., instead of prolactin. The results are given in Tables IV and V and in Fig. 3 and 4. It appears that the mammogenic growth response in hypophysectomised mice treated with steady doses of oestrone and progesterone together with variable doses of prolactin or growth hormone was proportional to the logarithm of the doses of the latter, for in both cases linear regression was

TABLE IV.—*Data Relating to Regression of Mammogenic Growth Responses on Dose of Prolactin in Hypophysectomised Mice Treated with Oestrone and Progesterone* 1·25 *μg. and* 7·5 *mg. per Mouse per* 5 *Days*

| Prolactin (mg./mouse/5 days) | Mammary growth response (Mean clubs/mouse) | Number of mice |
|---|---|---|
| 0·0008 | 13·76 | 13 |
| 0·004 | 20·53 | 13 |
| 0·02 | 46·77 | 13 |
| 0·1 | 61·92 | 13 |
| 0·5 | 65·75 | 12 |
| 2·5 | 101·45 | 11 |

Linear regression is highly significant $P < 0 \cdot 001$.

significant ($P < 0{\cdot}001$ and $P < 0{\cdot}01$ respectively). In incompletely hypophysectomised mice treated with oestrone and progesterone alone, it might therefore be possible to use the mammogenic growth response as a guide to the presence and amount of the circulating mammotrophic hormones which have been excreted by the residual pituitary fragments.

FIG. 4.—Regression of mammary growth response on dose of growth hormone in hypophysectomised mice treated with oestrone and progesterone.

TABLE V.—*Data Relating to Regression of Mammogenic Growth Responses on Dose of Growth Hormone in Hypophysectomised Mice Treated with Oestrone and Progesterone,* 1·25 *μg. and* 7·5 *mg. per Mouse per* 5 *Days*

| Growth hormone dose (mg./mouse/5 days) | Mammary growth response (Mean clubs/mouse) | Number of mice |
|---|---|---|
| 0·000032 | 21·00 | 13 |
| 0·00016 | 20·61 | 13 |
| 0·0008 | 25·53 | 13 |
| 0·004 | 22·84 | 13 |
| 0·02 | 59·76 | 13 |
| 0·1 | 65·07 | 13 |
| 0·5 | 97·46 | 13 |

Linear regression is statistically significant $P < 0{\cdot}01$.

Forty-three hypophysectomised mice injected with oestrone and progesterone in arachis oil were examined histologically for residual fragments of pituitary. The total dose of oestrone was 1·25 μg. and of progesterone 7·5 mg., one-tenth of this being given at each injection, night and morning, for 5 days. Nine mice were shown to have residual fragments of pars anterior, all of them size +.

The mean mammogenic growth response ± standard error in these mice was 6·78 ± 4·3 compared with 4·74 ± 1·401 in the remaining 34 which had no demonstrable pituitary fragments. The difference was not statistically significant

($P = 0{\cdot}1$). Each of these groups could be divided up, however, depending on whether the first injection was given 6 or less, or 7 or more, days after operation. In the latter case, the 6 mice having fragments of size + had a mean mammogenic growth response of $2{\cdot}5 \pm 1{\cdot}453$ while the remaining 22 with no demonstrable fragments had a mean mammogenic growth response of $1{\cdot}36 \pm 0{\cdot}520$. Once again the difference was not statistically significant ($P > 0{\cdot}2$).

A comparison was also made between mammogenic growth responses of *completely* hypophysectomised mice (*a*) having their first injection 6 days or less after operation (12 mice, mean M.G.R. $= 10{\cdot}92 \pm 3{\cdot}219$) and (*b*) having their first injection 7 days or more after operation (22 mice, mean M.G.R. $= 1{\cdot}36 \pm 0{\cdot}520$). The mice having a first injection a week or more after operation gave consistently low mammogenic growth responses, while those injected within a few days of operation not only gave high mammogenic growth responses but showed greater variation from one mouse to another. The difference between the two groups was very highly significant ($P < 0{\cdot}001$). It is possible that this difference might be due to the incomplete excretion or destruction of the mamotrophic hormone(s) during the first few post-operative days. If this is so, it means that in determining mammotrophic activity, false positives are liable to occur if hypophysectomised mice are used too soon after operation.

There is yet another way in which the mammary gland might be used in trying to assess the functional activity of fragments of anterior pituitary. Since the secretion of oestrogenic and progestational steroids is under the control of the pars anterior, and since oestrone + progesterone + impure prolactin has been shown to be actively mammogenic, it may be possible to estimate gonadotrophin activity indirectly by means of the mammogenic growth response in hypophysectomised mice treated with prolactin alone. Twenty-three hypophysectomised mice treated with 50 i.u. prolactin per mouse per 5 days were examined histologically for evidence of pituitary fragments. Fragments of size + were found in 6 mice. These had a mean mammogenic growth response of 1·16 compared to 1·35 for the 17 mice in which no fragments were found. It is clear that no measurable gonadotrophin activity could be detected by this method in mice with fragments of size +.

*Testis and seminal vesicle weights*

The direct measurements of gonadotrophin excretion in mice is not practicable but activity may be assessed indirectly by examination of the appropriate target organs. Sixty-nine mice were available for study. At death 7–16 days after operation, the testes and seminal vesicles were taken for weighing. In this group the 42 animals completely hypophysectomised had a mean testis weight ± standard error of 29·54 mg. ± 2·55 mg. with a mean seminal vesicle weight (41 animals) of 1·84 mg. ± 0·083 mg. ; the 6 mice with size + fragments had a mean testis weight of 39·32 mg. ± 12·424 mg. with a mean seminal vesicle weight of 2·27 mg. ± 0·249 mg. ; while the 21 mice with size +++ fragments (intentionally incomplete operations) had a mean testis weight of 74·92 mg. ± 7·83 mg., with a mean seminal vesicle weight of 19·11 mg. ± 3·166 mg.

In animals with size + fragments, testis weights did not differ significantly from those in completely hypophysectomised mice. Seminal vesicle weights, however, showed a greater difference. If it is admitted that complete hypophysectomy could only lead to a reduction in target organ weight, then the value of

$P < 0 \cdot 1$ could be halved to $P < 0 \cdot 05$, which is statistically significant. In mice with size +++ fragments both testis and seminal vesicle weights showed differences which were highly significant ($P < 0 \cdot 001$ in both cases).

*Adrenal weights*

Just as deprivation of gonadotrophin gives rise to atrophy of testes and seminal vesicles, so deprivation of adrenocorticotrophic hormone leads to atrophy of the adrenals. As in the examination of testis weights, 69 mice were available for the adrenal study. Forty-two completely hypophysectomised animals had a mean weight ± standard error of 1·17 mg. ± 0·022 mg.; 6 mice with size + fragments had a mean adrenal weight of 1·22 mg. ± 0·054 mg.; while the 21 mice with +++ fragments had a mean adrenal weight of 2·30 mg. ± 0·096 mg. No significant difference in mean adrenal weights was found between mice possessing size + fragments and those with none ($P > 0 \cdot 2$); however, there was a highly significant difference in the comparison of mice with size +++ fragments and those containing none ($P < 0 \cdot 001$).

### DISCUSSION

From a consideration of these results it is clear that the presence or absence of size +++ fragments was associated with very significant differences in somatic weight gain or loss, and also with significant differences in the weights of testes, seminal vesicles and adrenals. Unless they were left behind deliberately, fragments of size +++ were very uncommon. Their identification was, therefore, relatively unimportant but their presence was detected by daily weighings of the operated mice, since animals gaining weight regularly after the first few post-operative days have been found to possess fragments of this size (Fig. 5).

The discovery of mice with size + fragments was a more important problem, for these occurred more frequently. It appears that significant differences in somatic and seminal vesicle weights were present between mice containing size + fragments and those with none. The seminal vesicle weight difference was found a satisfactory indicator by Bahner and von Graff (1957) but it has the disadvantage that it entails either another operation, or death of the mouse. In the appraisal of mice for experiment the advantages lie clearly with selection by somatic weight. Unfortunately, the 156 mice we have studied in this connection, were too few in number to permit an accurate forecast of the level of weight increase to be confidently associated with the presence of size + fragments. It has become our habit, however, to discard as unsuitable any mouse exceeding its operation weight by 10 per cent on the seventh post-operative day.

Size + fragments appear to have been insufficiently active biologically to give a significantly increased mammogenic growth response with injections of oestrone and progesterone and this has apparently also been true of injections of prolactin given alone. Since fragments of size + are virtually the only size we have found in our mice, this implies that it is no longer necessary for us to cut serial sections of pituitary fossae from hypophysectomised mice treated with oestrone and progesterone, and used in the determination of mammogenic growth responses.

Occasionally no pituitary fragments were found in a mouse which appeared biologically to show evidence of pituitary activity as judged by weight gain following the operation, or a higher mammary growth response than was expected following oestrone + progesterone administration. There are several possible explanations :

1. The mouse might have been undernourished or partly dehydrated at the time of operation and gained weight subsequently to a normal level.

2. Fragments of pars anterior might have been left behind which continued to secrete hormones for a short time but did not survive permanently. Such fragments have, in fact, been occasionally observed (Young, 1959).

FIG. 5.—Mean somatic weight gain in " hypophysectomised " mice with fragments of pituitary, size + + +.

3. At the time of operation, viable fragments of pars anterior might have been transplanted into the brain, survived there and continued to secrete. Since our usual technique of serially cutting the pituitary fossa involved removal of the brain before embedding, it was unlikely that any such fragments would be found at subsequent examination. To test this possibility, serial sectioning was carried out on 63 mice without prior removal of the brain. Although fragments of pars anterior (+ and + + +), pars intermedia and pars posterior were present in some of these mice, in no single instance was a pituitary fragment found outside the pituitary fossa. Leaving the brain in place made an already tedious procedure more difficult, for the pituitary fossa could no longer be directly observed. The block of tissue had to be trimmed thicker than usual and many more sections were required to cut through it.

4. Finally, there was the possibility of the presence of a biologically active ectopic embryonal remnant. We have no information of its occurrence in mice.

It is interesting to note that there was no evidence of regeneration of size + fragments up to three months after the operation, and bony regeneration of the removed plate of basi-occiput was incomplete.

### SUMMARY

1. Over 200 hypophysectomised mice were examined histologically for the presence of residual fragments of pituitary. The presence or absence of pars anterior fragments was compared statistically with differences in somatic weight; testis weight; seminal vesicle weight and adrenal weight, and with the mammogenic growth response after treatment with oestrone and progesterone.

2. The presence or absence of size + fragments was associated with significant differences in somatic and seminal vesicle weights. Differences in testis weight and adrenal weight were not statistically significant.

3. It was not found possible to forecast accurately the presence of size + pars anterior fragments from a consideration of weight alone.

4. Mice with size + pars anterior fragments did not show significantly increased mammogenic growth responses when treated with oestrone 1·25 $\mu$g. and progesterone 7·5 mg. per mouse per 5 days.

5. In mice completely hypophysectomised and treated with oestrone 1·25 $\mu$g. and progesterone 7·5 mg. per mouse per 5 days the mammogenic growth response was significantly greater if injections were started less than 6 days after operation than if they were started 7 days or more after operation.

We are indebted to Dr. C. C. Spicer, Head of the Division of Statistics of the Imperial Cancer Research Fund, for advice in statistical methods, and to Messrs. J. Gilbert, T. O'Connor and P. V. Sharp for technical assistance.

We are grateful also to the Endocrinology Study Section of the National Institutes of Health, Bethesda, Maryland, U.S.A. for the gifts of growth hormone and prolactin, which have made this work possible.

### REFERENCES

Bahner, F. and von Graff, H.—(1957) *Acta endocr., Copenhagen*, **24**, 333.
Griffiths, M.—(1941) *J. Physiol.*, **100**, 104.
Hadfield, G. and Young, S.—(1956) *Brit. J. Cancer*, **10**, 324.
Lostroh, A. J. and Jordan, C. W.—(1955) *Proc. Soc. exp. Biol. N.Y.*, **90**, 267.
Thomas, F.—(1938) *Endocrinology*, **23**, 99.
Young, S.—(1959) *Brit. J. Cancer*, **13**, 208.